# Resistencia diurética e insuficiencia cardíaca: entre la congestión y la disfunción renal

**DOI:** 10.47487/apcyccv.v1i3.72

**Published:** 2020-09-30

**Authors:** Paola Morejón Barragán

**Affiliations:** 1 Instituto Argentino de Diagnóstico y Tratamiento. Buenos Aires, Argentina.

**Keywords:** Insuficiencia Cardíaca, Diuréticos, Síndrome Cardiorrenal, Heart Failure, Diuretics, Cardio-Renal Syndrome

## Abstract

La insuficiencia cardiaca (IC), uno de los principales contribuyentes de la morbimortalidad cardiovascular en la actualidad, nos enfrenta a grandes retos. La interacción riñón corazón tiene particular atención por el desarrollo del denominado síndrome cardiorrenal (SCR) y, con este, la resistencia a los diuréticos (RD), predictor de eventos adversos en IC aguda independiente de la tasa de filtrado glomerular (TFG). Su desarrollo es secundario a múltiples causas, por lo que se requiere una evaluación integral de todas ellas. En los últimos años ha tomado relevancia la congestión dentro del mecanismo fisiopatológico del SCR, por generar y perpetuar mutuamente el daño en estos dos órganos. Ante la importancia de la congestión, los diuréticos se mantienen como clave para el tratamiento, aunque su empleo ampliamente es empírico por la escasa evidencia disponible. El paradigma del tratamiento basado en evidencia es esquivo en este escenario, por lo que una pregunta permanece sin respuesta ¿intervenciones para tratar la RD o para prevenirla modifican el pronóstico en IC aguda?

## Abstract (en)

Nowadays, heart Failure (HF) is one of the main contributors of cardiovascular morbidity and mortality, this faces us with great challenges. The heart-kidney interaction receives particular attention due to the development of the so-called cardiorenal syndrome (CRS) and the diuretic resistance, latter is a predictor of adverse events in acute HF and is independent of the glomerular filtration rate. Development of diuretic resistance is secondary to multiple causes, so a comprehensive evaluation of all of them is required. In recent years, congestion has become relevant within the pathophysiological mechanism of CRS, as it mutually generates and perpetuates damage in these two organs. Given the importance of congestion, diuretics remain the cornerstone of treatment, although their use is largely empirical due to the limited evidence available. The evidence-based treatment paradigm is elusive in this scenario, so one question remains unanswered: Do the interventions to treat or to prevent the diuretic resistance modify the prognosis in acute HF?

La insuficiencia cardiaca (IC) constituye una pandemia del siglo XXI, cuya prevalencia está en aumento, en parte relacionado con la mayor sobrevida de la población, así como con el éxito farmacológico que hemos logrado en uno de sus fenotipos. Durante un episodio de descompensación, el 90% de los pacientes presenta signos de congestión **(**Figura 1**)** ^(^^1^, situación clínica que nos enfrenta diariamente a un reto: tratar la congestión sin descuidar el riñón.

**Figura 1:**
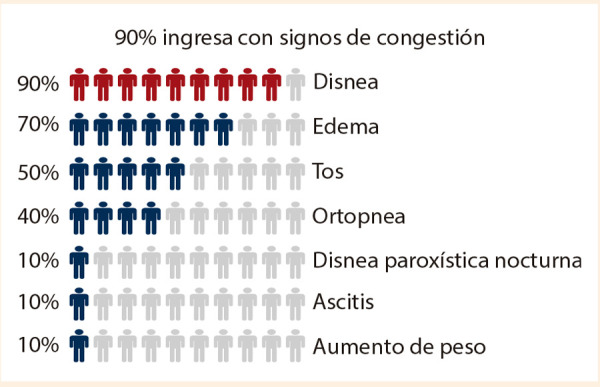
El sintoma mas prevalente durante las descompensaciones por IC es la congestion.

El vínculo entre el corazón y el riñón ha sido revisado extensamente en las últimas dos décadas. Aunque el impacto de las variables hemodinámicas es un acuerdo general, la visión ha cambiado. En la actualidad se conoce el menor impacto que tiene el gasto cardiaco, mientras que la congestión ha tomado relevancia; hemos pasado de la teoría de la falla anterógrada a la falla retrógrada ^2^^-^^4^. Al ser predominante la congestión durante la descompensación de la IC, los datos indican que los diuréticos de asa (fármacos para tratar la congestión), a pesar de no ser un grupo farmacológico que modifique la enfermedad y, por ende, tengan impacto en el pronóstico de los pacientes, son los fármacos centrales para el tratamiento de un evento agudo, son prescriptos hasta en un 90% de los casos, con similar porcentaje de pacientes que lo reciben al alta ^1^^,^^5^. Uno de los cuestionamientos al que nos enfrentamos diariamente al momento de tratar un episodio de descompensación de IC, es la interpretación de la función renal, puntualmente, de las fluctuaciones de la creatinina.

Históricamente la interpretación de la función renal en este escenario ha sido simplificada a la visión reduccionista del valor de la creatinina, consideremos que la utilizamos para calcular la tasa de filtrado glomerular estimada (TFGe), es decir, si el valor de creatinina aumenta, la función renal empeora y viceversa. Esto nos ha llevado a considerar erróneamente que, a pesar del tratamiento exitoso de la congestión, un descenso transitorio de la TFGe estaría asociado con peor pronóstico. Sin embargo, a la luz de la literatura, es probable que la TFG sea un mal marcador para evaluar la respuesta y la eficiencia diurética; ante lo expuesto, es pertinente considerar que se requiere una redefinición de la disfunción renal en el marco de la IC aguda ¿estamos seguros que existe lesión renal? ^6^^,^^7^.

Parece que el término de eficiencia diurética (ED) que integra el balance hídrico, los cambios en el peso o natriuresis, por miligramos de furosemida administrada, es el más apropiado para calificar la respuesta a la terapia diurética. Sus limitantes, no tiene umbral establecido y no integra el uso concomitante de otros diuréticos. Con estas consideraciones podemos inferir que, si en algún momento estos fármacos dejan de ser eficientes, la resistencia a los diuréticos (RD) está presente. La falta de consenso para definir la RD ha generado que su incidencia no sea establecida, no obstante, impresiona estar en aumento.

El principal objetivo de esta revisión es resumir los mecanismos fisiopatológicos del amplio espectro de la ED, que nos acerca a la RD en IC y al síndrome cardiorrenal (SCR), así como brindar una visión de su tratamiento basada en la comprensión de la compleja fisiopatología que rodea esta interacción. Refiriéndonos en todo el texto a la IC aguda.

## Definiendo el problema

Se han planteado varias definiciones empleando un parámetro métrico **(**Tabla 1**)**, todas ellas tienen como base la falta de respuesta al tratamiento antes que el problema biológico de base que desencadena la RD; a esto se suman las dificultades propias de la medición de estos parámetros y sus constantes variaciones. Entre las variables que han sido consideradas, tenemos:

**Tabla 1:** Diferentes definiciones de RD que utilizan un parámetro métrico

Parámetro	Definición
Pérdida de peso ^10^	0-2,7 kg por 40 mg de furosemida o equivalentes.
Diuresis ^10^	< 1400 mL por 40 mg de furosemida o equivalentes.
Excreción fraccional de sodio ^17^	< 0,2%
*Concentración de sodio urinario/concentración de furosemida urinaria ^25^	< 2 mmol/mEq/L
*Sodio urinario ^26^	< 50-70 mmol/L

*Muestra de orina de una única evacuación; RD: resistencia a los diuréticos

● La pérdida de peso ^8^^-^^10^ y el balance negativo total ^11^^,^^12^: Estas medidas son imprecisas, de difícil cuantificación exacta y en las que influyen otros factores. Además, la pérdida de peso es un mal predictor de euvolemia; esta última tampoco ha sido estandarizada. Perder poco peso ≤ 1kg, y subir de peso > 1 kg, se asocian a mal pronóstico. Por cada kilogramo de peso que sube un paciente durante la internación, el riesgo de mortalidad a 30 días u hospitalización por IC, se incrementa 16% ^13^^,^^14^.

● El peso y la diuresis: tienen una débil correlación entre sí. Sin olvidar que la diuresis por sí sola, nos habla del volumen de orina, pero si no vemos su composición omitimos el principal generador de la sintomatología de la congestión: el sodio ^10^^,^^12^.

● La natriuresis ^15^ varía ampliamente. Aunque nos brinda una pauta de la tasa de descongestión, padece las mismas limitaciones al medirlo en orina de 24 horas. Por esta razón se ha considerado la medición de sodio en orina recolectada sea a 1-2 horas después de recibir furosemida. De ser baja la concentración urinaria de sodio a las seis horas del inicio de la terapia diurética, es un marcador que se asocia con mayor mortalidad (HR: 3,81; IC 95%: 1,92-7,57; p < 0,001). Ha generado expectativa la medición de sodio en una única muestra de orina; su limitación, no incorporar la dosis del diurético; su ventaja, brinda un valor de referencia (50-70 mEq/L), por debajo del cual se esperarían malos resultados, es decir, le daría el umbral que carece la definición de la ED ^16^^,^^17^ y, por último;

● La excreción fraccional de sodio: parámetro que parece ser más preciso en pacientes que tienen disfunción renal de base ^18^^,^^19^.

Estas variables no han sido estandarizadas y sus puntos de corte no han sido validados; es decir, nos aproximamos, pero seguimos sin parámetro métrico establecido; aunque la medición de sodio en una muestra aislada por su versatilidad se perfila como el parámetro requerido en la definición de eficiencia diurética, se requieren estudios que nos confirmen su utilidad ^20^^,^^21^. Esta falta de consenso nos ha llevado a mantener una definición cualitativa, la RD es la inadecuada respuesta diurética / natriurética a un régimen diurético escalonado y adecuado, el cual no permite lograr descongestión efectiva ^22^. Cabe mencionar que este concepto excluye la euvolemia, ya que, al llegar a la meta, la diuresis disminuye y no por falta de respuesta, si no como secuencia de reducción del volumen intravascular, es decir, es una respuesta fisiológica.

Definitivamente, la RD es el reto de la definición de lo adecuado. Empecemos por la diuresis adecuada. Un volumen de diuresis de 3-4 L por cada 40 mg de furosemida, se ha establecido como respuesta normal en personas sanas, en pacientes con IC el panorama cambia, siendo la respuesta a diuréticos incluso menor que la de los pacientes con enfermedad renal crónica ^8^^,^^23^. No es lo mismo lograr una diuresis de 500 mL con 40 mg de furosemida, que 100 mL con la misma dosis; como ya se mencionó, uno de los problemas en la definición es no especificar el uso de otros diuréticos; sin embargo, nuestro objetivo no se modifica, lo importante es lograr diuresis y natriuresis, y esto va a depender de un régimen adecuado ^11^. Establecer el régimen diurético adecuado no es una tarea sencilla, no hay un valor absoluto (dosis de fármacos) para definirlo, y se ve influenciado por factores como: dosis, frecuencia, requerimiento de otros diuréticos y la respuesta previa al fármaco empleado. Los reportes indican que entre el 20-50% ^24^^,^^25^ de pacientes presentan una mala respuesta inicial a diuréticos intravenosos, lo que lleva a considerarlos, como resistentes a los diuréticos. Así, la ED disminuida es una aproximación a la RD, esta última impresiona ser más relativa. Estandarizar su definición debería ser el objetivo de futuras investigaciones, necesario para homogenizar los criterios de inclusión en ensayos clínicos. No solo es importante definirla, se requiere conocer los factores de riesgo que pueden desencadenarla y, por ende, quizás intervenir para prevenirla; estos factores incluyen: hipotensión arterial, urea elevada, IC de etiología isquémica y diabetes ^8^^,^^9^.

La RD ha sido identificada como factor independiente de resultados adversos como: empeoramiento de la IC durante la hospitalización, internación prolongada, predictor de mortalidad temprana postalta y de rehospitalización ^8^^,^^9^^,^^11^. En el estudio ESCAPE, la baja ED se asoció con incremento de la mortalidad (HR: 3,57; IC 95%: 1,46-8,73; p= 0,005), peor aun si era en presencia de altas dosis de diuréticos de asa ^11^. Es imperioso tomar en cuenta que el problema no son las dosis altas de diuréticos, el problema es la RD, esta reinterpretación de conceptos nos ayuda a comprender que equívocamente hemos relacionado a la furosemida con malos resultados sin considerar que el problema está en la falta de respuesta, en la necesidad de las altas dosis y no en el fármaco *per se*.

## Identificando el inicio del problema desde el mecanismo de acción de los diuréticos de asa

**Figura 2:**
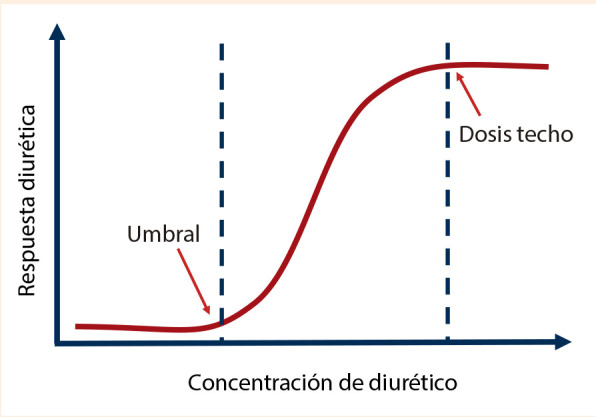
Curva dosis respuesta de los diureticos de asa.

Para que la RD se instaure, más allá del progreso de la enfermedad, tenemos que tomar en cuenta lo insistentes que debemos ser al considerar el régimen de tratamiento adecuado, a continuación, revisaremos algunas omisiones durante el uso de diuréticos de asa:

1. Dosis insuficiente: los diuréticos de asa tienen una curva dosis-respuesta muy pronunciada, en forma de S itálica. Los pacientes con IC presentan menor respuesta diurética, incluso menor que los pacientes con enfermedad renal crónica **(**Figura 2**)** ^(^^26^, por lo que, iniciar con la dosis adecuada para lograr el umbral, es más importante que la frecuencia de su uso ^6^^,^^22^^,^^26^. Si bien en el estudio DOSE ^27^, el brazo «dosis baja» recibió por vía intravenosa (IV) la misma dosis que recibía por vía oral (VO), es decir, se consideró equivalencia 1:1 (este detalle se encuentra en el protocolo del estudio); realmente este grupo recibió una dosis duplicada, ya que, debemos recordar que la equivalencia conocida es del 50 % para la dosis IV en relación a la dosis por VO. Bajo este mismo razonamiento se comprenderá que los pacientes del brazo «dosis alta» recibieron quintuplicada su dosis habitual; por lo tanto, para conseguir la concentración sérica adecuada que logre el umbral de respuesta, es mejor una dosis alta, recomendación validada por el estudio DOSE.

Un dato que suele pasar desapercibido es que la respuesta diurética a la dosis sigue una escala logarítmica y no lineal, por lo que es necesario cuando se realicen incrementos de dosis de furosemida, duplicar directamente la dosis ^22^^,^^26^.

2. Retraso en el inicio de la terapia sin considerar el beneficio de la venodilatación: los beneficios de furosemida específicamente van más allá de la diuresis, su efecto inmediato a los 5 min es la venodilatación, lo cual mejora la tasa de rellenado transcapilar; mientras que el efecto diurético se logra luego de 20-30 min de la administración ^28^; es decir, aliviamos primero los síntomas y después eliminamos volumen.

3. Omitir un bolo previo al inicio de la infusión continua de furosemida o no administrar bolos con la frecuencia adecuada, es erróneo. ¿Cuál es la mejor forma de administrar diuréticos de asa? Esta pregunta ha sido un debate frecuente. Mantener de forma constante la concentración sérica del diurético por sobre el nivel umbral (infusión continua) no ha demostrado ser superior a la administración en bolos, según los resultados del estudio DOSE ^27^. Sin embargo, evidencia complementaria ha relacionado al uso en infusión continua con mayor diuresis y mayor reducción de péptidos natriuréticos ^29^. No obstante, surgen dos particularidades en relación al estudio DOSE: 1) Los pacientes que recibieron furosemida en bolos presentaron de manera significativa el doble de posibilidades de requerir dosis más altas y otro diurético asociado, en comparación con el grupo de infusión continua; y 2) el brazo de infusión continua no recibió bolo inicial, lo cual acorde con la revisión de Ellison y Felker ^26^, sería un posible factor determinante de los resultados que obtuvo este estudio. Tomando en cuenta que en infusión continua la respuesta es tardía, ya que para lograr el nivel plasmático adecuado y mantener la dosis estable deben pasar entre 6 y 10 h, toma importancia una dosis en bolo inicial, la cual cubriría este período de tiempo; por lo que se recomienda iniciar la infusión continua con un bolo, o cuando se incremente la dosis administrar un bolo previo. Mientras que, en caso de administrar bolos de furosemida, la frecuencia cobra importancia y hacerlo cada 6-8 h sería un tiempo recomendable para mejores resultados ^26^. Otros efectos que hay que poner sobre el tablero se relacionan con la posible mayor actividad neurohormonal relacionada al uso de infusión continua. Al momento, seguimos sin respuesta basada en evidencia.

## La fisiopatología nos orienta en el tratamiento en tiempos actuales

**Figura 3:**
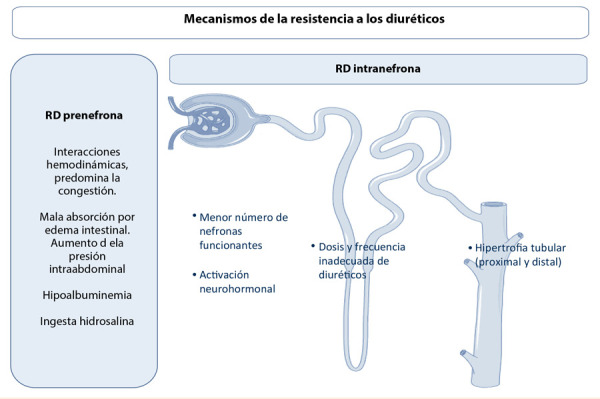
Mecanismos de la resistencia a los diureticos (basado en el concepto del experto Prof. J. Testani)

Basándonos en el concepto de expertos, el profesor Jeffrey Testani propone reconocer dos grandes mecanismos que generan RD: pre e intranefrona **(**Figura 3**)**.

### Mecanismos de RD prenefrona

Históricamente se creyó que el bajo gasto cardíaco era el mecanismo principal para desencadenar el SCR y la RD, sin embargo, literatura reciente no apoya esta hipótesis y propone como principal contribuyente a la congestión venosa; basándose en la disminución del gradiente de presión arteriovenoso en el glomérulo, así como también por el retraso que se genera en la absorción de fármacos debido al edema intestinal ^2^^-^^4^. Es importante considerar que el uso de diuréticos es eficiente cuando el volumen intravascular está incrementado, caso contrario, su depleción fomentaría mayor activación neurohormonal ^26^.

En este mecanismo encontramos al bajo gasto, cobrando importancia la teoría de los vasodilatadores que, por ende, lo incrementarían, esta prueba de concepto no ha dado resultados positivos y ha fallado en demostrar incremento de la diuresis ^30^.

En cuanto a la hipoalbuminemia, bajo la teoría que el 90% de los diuréticos de asa se unen a esta y dicha condición generaría no solo menor volumen intravascular para generar diuresis sino menor disponibilidad del fármaco, la literatura que pone a prueba esta hipótesis en IC es escasa; un pequeño estudio evaluó la administración concomitante de albúmina IV más furosemida, sin resultados positivos en diuresis total o dosis de diurético requerida ^31^.

A la fecha, la indicación de la dieta hiposódica carece de evidencia suficiente ^1^. No obstante, literatura disponible sugiere que a mayor natriuresis, incrementar la ingesta de sodio durante la descompensación resultaría beneficiosa ^22^^,^^26^. Esta sería la base para el uso de la solución salina hipertónica. Aunque la calidad de la evidencia no es la esperada, sus efectos son positivos a corto plazo, a través del incremento de la ED y mayor pérdida de peso, es decir, nos permite lograr el objetivo inmediato: descongestionar ^32^^,^^33^. Los beneficios a largo plazo no son consistentes, por lo que se requiere de estudios diseñados para probar que sus resultados no se basen en mitigar los efectos de conductas médicas como la restricción de sodio a través de la dieta, recordemos que los trabajos de suero hipertónico reportan dieta normosódica. Sin mencionar que olvidamos el otro componente de la sal: el cloro, reponer lo que restringimos y salir del sodio como centro de la retención hídrica es otro punto a considerar en la IC y la RD.

### Mecanismos de RD intranefrona

Los mecanismos a través de la nefrona son diversos. La respuesta a los diuréticos está limitada por la cantidad de nefronas funcionantes, las cuales en sí representan la función renal, la cual se estima mediante la TFG. Sin embargo, de manera independiente, la pérdida de nefronas disminuye la cantidad de diuresis, a pesar que las nefronas aún funcionantes mantengan la excreción de sodio ^23^^,^^26^. Por lo tanto, la TFG tiene una mala relación con la pérdida hídrica y la ED, el paciente puede orinar en menor cantidad, pero conservar la natriuresis ^11^. Así, la disfunción renal *per se* (pérdida de nefronas), resulta relevante en los pacientes con enfermedad renal crónica siendo menor su impacto en pacientes con IC aguda.

Evidencia reciente sugiere que defectos a nivel tubular son más importantes que la escasa entrega de los diuréticos de asa a su sitio de acción ^19^. Otros de los mecanismos intranefrona constituyen: la activación neurohumoral ^23^, tradicionalmente relacionada con la RD, así como la acción de los diuréticos en el asa de Henle, lo cual fue abordado en la sección previa al mencionar la necesidad de dosis y frecuencia adecuada.

Finalmente, si consideramos un mecanismo adaptativo inicial, la hipertrofia del sistema tubular, la cual se genera por la exposición continua a los diuréticos, según experimentos animales, determinaría la hiperfunción de los túbulos. Basándose en este mecanismo, el remodelado de la nefrona sería considerado como objetivo terapéutico con fármacos que actúen tanto a nivel de túbulo proximal como del distal; al ser el responsable de la hiperfunción y, por ende, mayor reabsorción de sodio, bloquear la acción de los túbulos parece un camino sensato ^23^^,^^26^, es decir, nos referimos al bloqueo secuencial de la nefrona. Recientemente se publicó el estudio 3T ^34^, ensayo realizado en un único centro, prospectivo randomizado, doble ciego, que evaluó la eficacia de tres diuréticos: metolazona vía oral, clorotiazida IV y tolvaptán vía oral, como terapia coadyuvante de furosemida, en 60 pacientes con RD, quienes antes del enrolamiento recibían una dosis promedio de 612 mg de furosemida en 24 h, con diuresis de 1188 mL en las primeras 12 h. A las 48 h se evaluó el efecto del segundo diurético administrado, sin diferencias significativas en la pérdida de peso y la diuresis total, aunque los pacientes tratados con clorotiazida fueron los que menos respondieron en estos parámetros, los tres grupos farmacológicos mejoraron la ED. Aunque no hay un claro ganador, y seguimos sin respuesta del segundo a bordo después de furosemida, el 3T es un ejemplo que, metodológicamente, es posible realizar estudios en este campo.

Bajo las consideraciones de los mecanismos involucrados en la RD, tomemos en cuenta varios principios al momento de decidir la terapia diurética idónea:

1. Asegurar dosis y frecuencia adecuada desde un inicio.

2. Evaluar tempranamente la conducta terapéutica inicial, a través de diuresis/natriuresis.

3. Maximizar las dosis de diuréticos de asa duplicando inmediatamente la dosis, antes de considerar el bloqueo secuencial, nuevamente evaluar la respuesta de manera oportuna es fundamental.

4. Combinar otros grupos farmacológicos según se considere el mecanismo principal que lleva a la ED disminuida, sin olvidar que el bloqueo secuencial no está libre de efectos adversos y que aún desconocemos el segundo fármaco y el momento ideal ^35^.

Hasta que más evidencia esté disponible, la decisión de los médicos para tratar a estos pacientes debe basarse en aspectos fisiopatológicos y mediante la reinterpretación de la literatura para tomar mejores decisiones. A la fecha, varios estudios están en curso para brindarnos respuestas.

## ¿Y la creatinina?

Los lectores podrán identificar que no hemos mencionado a la creatinina dentro de los párrafos previos. Basándonos en la literatura publicada, este biomarcador no es el indicado por sí solo para evaluar la función renal, y menos aun que se lo considere como determinante único para la toma de decisiones, específicamente, detener terapias de descongestión e incluso fármacos que han demostrado beneficio en mortalidad. A continuación, haremos una breve mención de lo planteado.

La mayoría de los pacientes con IC presentan algún grado de disfunción renal, lo que incrementa su riesgo de mortalidad hasta en un 50%. Un análisis del registro ADHERE identificó que aproximadamente el 60% de pacientes presenta disfunción renal al menos de grado moderado ^36^, esta situación predispone a mayor riesgo de desarrollar lesión renal aguda (LRA) durante un episodio de IC descompensada, cuya prevalencia se estima entre el 20 al 70% ^37^. Ante la importancia de identificar a los pacientes que desarrollarán LRA, de manera arbitraria se ha considerado a la creatinina como el biomarcador «ideal» para este fin. Si bien la LRA se asocia con mayor mortalidad, especialmente en pacientes con ERC, subanálisis de diferentes estudios como el ESPACE, SOLVD, PROTECT, entre otros ^38^^,^^39^, han demostrado que fluctuaciones aisladas de la creatinina carecen de implicancias en el pronóstico, por el contrario, bajo el enfoque de la hemoconcentración, estas elevaciones se han asociado con menor mortalidad. Estas fluctuaciones deben ser consideradas respuestas fisiológicas a la descongestión, aun más si se acompañan de mejoría clínica, por lo que no deben ser consideradas para suspender dicha intervención. Por otro lado, cabe mencionar que el incremento sostenido de la creatinina que se acompaña de congestión persistente sí ha demostrado asociarse con peor pronóstico y debería ser el gatillo para buscar otra intervención. Una vez más, la congestión es la clave ^4^^,^^7^.

Considerar que un marcador de libre filtración por el glomérulo, que puede ser excretado por el túbulo sin que esto indique lesión tubular *per se*, con factores externos que alteran su producción y eliminación, tenga la capacidad de predecir la función renal, parece poco apropiado. Es momento de buscar e identificar marcadores alternativos para evaluar la función renal de manera precisa, y reconsiderar si realmente nos enfrentamos a lesión renal durante un episodio agudo de IC; la evidencia al momento no sugiere que se genere un daño estructural, el replanteamiento de la interpretación se basa en reconocer que el verdadero factor pronóstico es la función renal basal más no la creatinina.
